# Associations between cardiometabolic index with kidney stones: evidence from NHANES 2007-2018

**DOI:** 10.3389/fendo.2025.1485477

**Published:** 2025-03-10

**Authors:** Suquan Zhong, Guoliang Li, Chao Tian, Maolin Jiang, Dong Chen, Hangtao Wang, Pengfei Diao

**Affiliations:** Department of Urology, Yuebei People Hospital Affiliated to Medical College of Shantou University, Shaoguan, China

**Keywords:** CMI, kidney stone, metabolic syndrome, cross-sectional study, NHANES

## Abstract

**Background:**

Kidney stones are a prevalent disorder that is linked to a range of metabolic variables. The cardiometabolic index (CMI) is a newly developed combined measure used to evaluate the state of cardiometabolic health. Nevertheless, the association between CMI and kidney stone remains little investigated.

**Methods:**

In this study, cross-sectional analysis was done on NHANES data from 2007 to 2018. The association between the prevalence of kidney stones and the CMI was investigated using a logistic regression analysis. To look into the nonlinear link between them, we used restricted cubic spline (RCS) analysis. The study was made more reliable and accurate by using sensitivity analysis and subgroup analysis to look for factors that may have contributed to the different results.

**Results:**

A significant association was seen between increased CMI and the prevalence of kidney stones (OR = 1.19, 95% CI: 1.06-1.32). The RCS analysis revealed crucial CMI values that exhibited a robust association within a certain range. Subgroup studies revealed that this link was particularly prominent among those below the age of 50, females, obese, CKD and diabetes patients. The dependability of the study’s conclusions was further established using sensitivity analysis.

**Conclusion:**

This study established a notable association between CMI and a higher prevalence of kidney stones, emphasizing the significance of CMI as a comprehensive measure for evaluating metabolic risk. Furthermore, it suggests that monitoring CMI levels could be beneficial in identifying populations with a high prevalence of kidney stones.

## 1 Introduction

Kidney stones are a prevalent urological ailment defined by the formation of crystalline deposits in the kidneys (1). Kidney stones occurrence and frequency have been increasing worldwide, presenting a significant public health issue (2, 3). Kidney stones may lead to severe discomfort and distress, perhaps causing urinary tract infections, hydronephrosis, and the progression of chronic kidney disease and renal failure (4). The recurrent reoccurrence of kidney stones generally requires many medical procedures, hence exacerbating the healthcare cost (5).

Traditionally, dietary habits, fluid intake, and genetic predisposition have been recognized as the primary risk factors for kidney stones (6, 7). However, there is a growing body of research exploring the connection between metabolic disorders and formation of kidney stone (8, 9). The CMI is a novel metric that assesses an individual’s cardiovascular and metabolic risk by integrating the waist-to-height ratio (WHtR) and the ratio of triglyceride (TG) to high-density lipoprotein cholesterol (HDL-C) (10–13). Elevated CMI values are often linked to metabolic disorders such as metabolic syndrome, obesity, and insulin resistance, which have been associated with kidney stone formation (10, 11). Metabolic problems may produce irregular excretion of calcium, oxalate, and uric acid in the urine, which increases the prevalence of kidney stones (3, 9).

This study examined the relationship between the prevalence of kidney stones and CMI by analyzing data from the National Health and Nutrition Examination Survey (NHANES). Our study aimed to investigate the potential correlation between CMI and kidney stones by analyzing a large and representative sample. The objective of this investigation is to offer new perspectives on the prevention and treatment of kidney stones.

## 2 Methods

### 2.1 Study population

This research used the NHANES database to do a cross-sectional analysis in order to evaluate the health and nutritional situation of the U.S. population completely. All NHANES participants provided written informed assent. The study encompassed 59,842 participants and collected data from 2007 to 2018. Age below 20 years, pregnancy status, incomplete kidney stone data, and incomplete CMI data (including waist circumference (WC), body height (BH), HDL-C, and TG) were the exclusion criteria.

### 2.2 Definition of exposure and outcome variables

The following method was used to figure out the exposure variable, CMI: WHtR = WC (cm)/BH (cm); CMI = WHtR × [TG (mmol/L)/HDL-C (mmol/L)]. The outcome variable was a yes answer to the question “Have you ever had kidney stones?” This was a history of kidney stones that the person themselves recorded. Previous study has shown that this self-reported history is accurate (14, 15).

### 2.3 Selection of covariates

In order to enhance the precision of the analysis and account for potential influencing factors, the study incorporated the following variables: age, sex, race, body mass index (BMI), educational attainment, ratio of income to poverty (PIR), serum uric acid levels, total cholesterol levels, smoking status, physical activity level, presence of diabetes, presence of chronic kidney disease (CKD), and presence of coronary artery disease (CAD). The BMI categories were classified as underweight (<18.5), normal (18.5-25), and obese (>25). The questionnaire’s self-reported diagnoses of heart disease or angina were used to determine CAD. Metabolic equivalent (MET) was employed to evaluate physical activity, with MET values below 600 indicating inactivity and MET values above 600 indicating activity. Diabetes and chronic renal disease were determined using self-reported medical histories.

### 2.4 Statistical analysis

The appropriate weights were employed to analyze the data from the NHANES survey. The CMI data was subjected to a logarithmic modification due to its left-skewed distribution. In order to evaluate the relationship between renal stones and log-transformed CMI, logistic regression analyses were implemented. Furthermore, the model incorporates a multitude of correctional factors. Nevertheless, no covariates were incorporated into model 1. The variables of age, sex, and race were considered in Model 2. The Model 3 was regulated for age, sex, race, BMI, PIR, education level, physical activity status, smoking, CAD, CKD, diabetes, blood uric acid, and total cholesterol. In order to undertake a more comprehensive analysis of the impacts of different CMI levels, the continuous variable of CMI was converted into a categorical variable. The relationship between kidney stones and CMI was established by using restricted cubic splines (RCS). The study conducted subgroup analysis to identify potential variables that might have impacted the outcomes. In order to assess the reliability and veracity of the findings obtained from this investigation, sensitivity studies were implemented. The R program was employed to conduct the statistical analyses, and statistical significance was determined by a P value of less than 0.05.

## 3 Results

### 3.1 Participant selection and baseline characteristics

**Figure 1:**
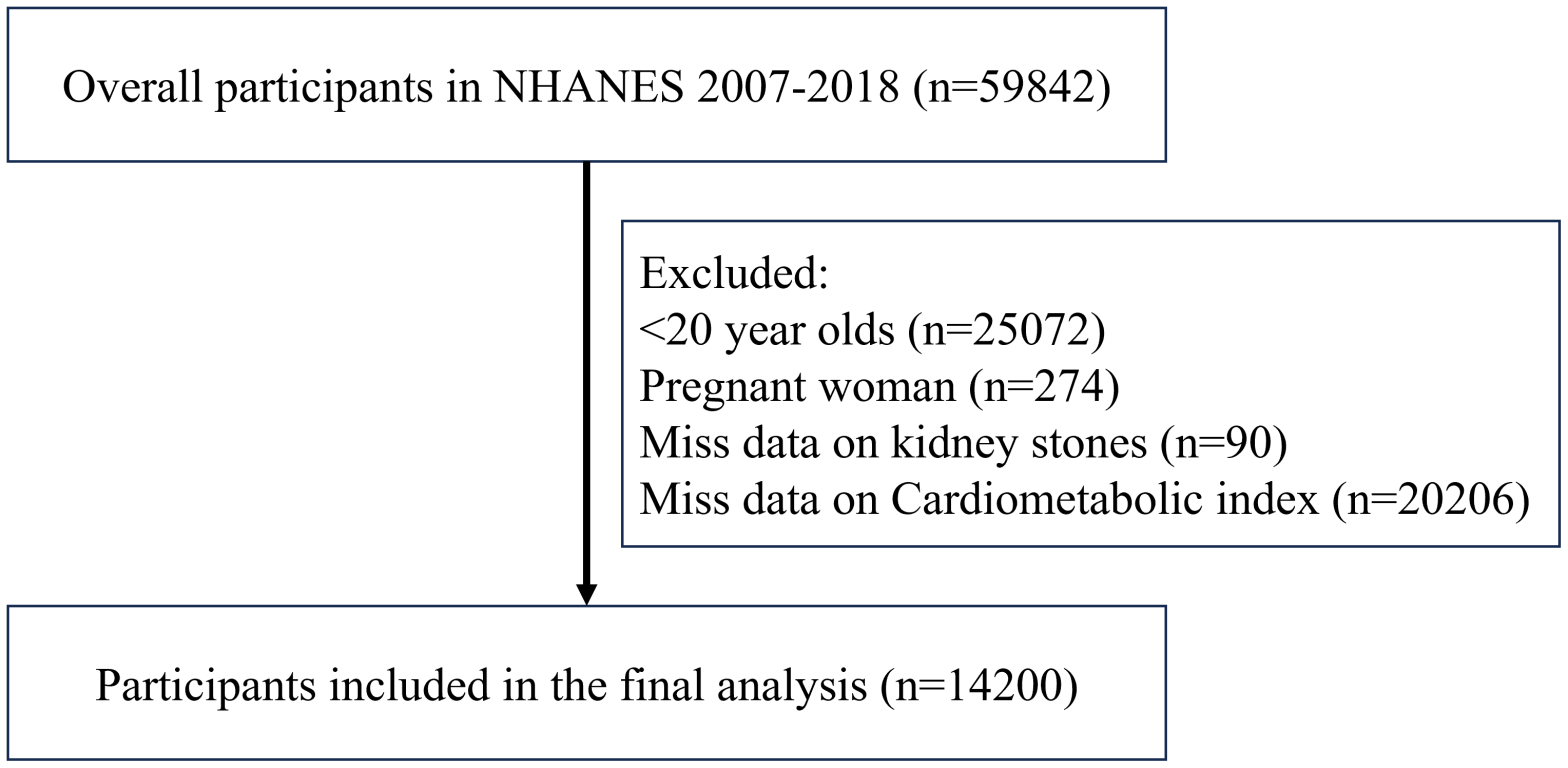
Includes participants in the process.

**Figure 1** illustrates the procedure of selecting and including individuals, and the research finally recruited 14,200 people. Out of the total, 12,843 individuals were not affected by kidney stones, however 1,357 individuals had kidney stones. The investigation revealed that those who were aged, male, non-Hispanic white, obese, smokers, and physically sedentary had a higher likelihood of developing kidney stones. Moreover, these individuals were prone to a heightened risk of acquiring diabetes, CAD and CKD. It is crucial to note that the CMI of individuals with kidney stones was significantly greater than that of persons without kidney stones. **Table 1** presents the initial characteristics of the participants.

**Table 1:** Baseline characteristics of the study population.

Characteristic	Group	Overall	Non-Kidney stones	Kidney stones	P value
n		14200	12843	1357	
Age (%)	<50	7274 (51.2)	6768 (52.7)	506 (37.3)	<0.001
	>50	6926 (48.8)	6075 (47.3)	851 (62.7)	
Sex (%)	Female	7265 (51.2)	6655 (51.8)	610 (45.0)	<0.001
	Male	6935 (48.8)	6188 (48.2)	747 (55.0)	
Race (%)	Mexican American	2178 (15.3)	1998 (15.6)	180 (13.3)	<0.001
	Non-Hispanic black	2843 (20.0)	2685 (20.9)	158 (11.6)	
	Non-Hispanic white	5873 (41.4)	5145 (40.1)	728 (53.6)	
	Others	3306 (23.3)	3015 (23.5)	291 (21.4)	
Education level (%)	Under high school	3540 (24.9)	3188 (24.8)	352 (25.9)	0.520
	High school	3192 (22.5)	2891 (22.5)	301 (22.2)	
	Above high school	7454 (52.5)	6750 (52.6)	704 (51.9)	
	No record	14 (0.1)	14 (0.1)	0 (0.0)	
BMI (%)	Underweight	242 (1.7)	235 (1.8)	7 (0.5)	<0.001
	Normal	3944 (27.8)	3682 (28.7)	262 (19.3)	
	Obese	9999 (70.5)	8911 (69.5)	1088 (80.2)	
Smoke (%)	No	7875 (55.5)	7183 (55.9)	692 (51.0)	0.002
	Yes	6313 (44.5)	5649 (44.0)	664 (48.9)	
	No record	12 (0.1)	11 (0.1)	1 (0.1)	
Activity status (%)	Active	7462 (52.5)	6811 (53.0)	651 (48.0)	<0.001
	Inactive	6738 (47.5)	6032 (47.0)	706 (52.0)	
CAD (%)	No	13178 (92.8)	12000 (93.4)	1178 (86.8)	<0.001
	Yes	1022 (7.2)	843 (6.6)	179 (13.2)	
CKD (%)	No	13721 (96.6)	12462 (97.0)	1259 (92.8)	<0.001
	Yes	459 (3.2)	365 (2.8)	94 (6.9)	
	No record	20 (0.1)	16 (0.1)	4 (0.3)	
Diabetes (%)	No	11969 (84.3)	10967 (85.4)	1002 (73.8)	<0.001
	Yes	1872 (13.2)	1564 (12.2)	308 (22.7)	
	No record	359 (2.5)	312 (2.4)	47 (3.5)	
Kidney stones (%)	No	12843 (90.4)	12843 (100.0)	0 (0.0)	<0.001
	Yes	1357 (9.6)	0 (0.0)	1357 (100.0)	
SUA (mean (SD)) (mg/dL)		5.51 (1.44)	5.49 (1.43)	5.66 (1.49)	<0.001
WC (mean (SD)) (cm)		99.32 (16.29)	98.75 (16.17)	104.67 (16.45)	<0.001
BH (mean (SD)) (cm)		167.13 (10.08)	167.11 (10.06)	167.34 (10.23)	0.414
TG (mean (SD)) (mmol/L)		1.41 (1.26)	1.40 (1.26)	1.54 (1.20)	<0.001
HDL (mean (SD)) (mmol/L)		1.39 (0.41)	1.40 (0.42)	1.31 (0.38)	<0.001
TC (mean (SD)) (mg/dL)		191.63 (41.54)	191.96 (41.73)	188.46 (39.61)	0.003
CMI (mean (SD))		0.76 (1.13)	0.75 (1.13)	0.92 (1.11)	<0.001

Mean (SD) for continuous variables, % for categorical variables. BH, Body height; BMI, Body mass index; CAD, Coronary artery disease; CKD, Chronic kidney disease; CMI, Cardiometabolic index; SUA, Serum uric acid; TC, Total cholesterol; TG, Triglyceride; WC, Waist circumference.

### 3.2 Association between CMI and kidney stones

The skewness of the CMI data was corrected by using a logarithmic adjustment. Log-transformed CMI and kidney stones are correlated, as shown in **Table 2** by logistic regression analysis. Kidney stones and log CMI showed a positive connection in Model 1 (OR=1.41, 95% CI=1.29-1.53). Once numerous variables were taken into account, the prevalence of kidney stones rose by 19% for every unit rise in log CMI (OR=1.19, 95% CI=1.06-1.32). Renal stones and increased CMI continued to be positively correlated even when CMI was changed from a continuous to a categorical category. Based on these findings, it seems that CMI may be a useful metric to assess kidney stone prevalence.

**Table 2:** Relationship between CMI and kidney stones.

		Model 1 OR (95%CI) P value	Model 2 OR (95%CI) P value	Model 3 OR (95%CI) P value
Kidney stones	log CMI	1.41 (1.29, 1.53) <0.001	1.34 (1.23, 1.46) <0.001	1.19 (1.06, 1.32) 0.003
	Q1	[Reference]	[Reference]	[Reference]
	Q2	1.41 (1.09, 1.84) <0.001	1.33 (1.02, 1.72) 0.033	1.16 (0.87, 1.55) 0.300
	Q3	1.88 (1.45, 2.44) <0.001	1.73 (1.32, 2.25) <0.001	1.40 (1.03, 1.91) 0.034
	Q4	2.20 (1.74, 2.78) <0.001	1.94 (1.53, 2.46) <0.001	1.46 (1.08, 1.97) 0.013
	P for trend	<0.001	<0.001	0.015

CI, Confidence interval; CMI, Cardiometabolic index; OR, Odds ratio; Q, Quartiles.

Model 1: No covariates adjusted; Model 2: Adjusted for Age, Sex, and Race; Model 3: Adjusted for Age, Sex, Race, BMI, PIR, Educational level, Smoke, Activity status, CAD, CKD, Diabetes, SUA, BMI, TC.

### 3.3 Non-linear association between CMI and prevalence of kidney stones

**Figure 2:**
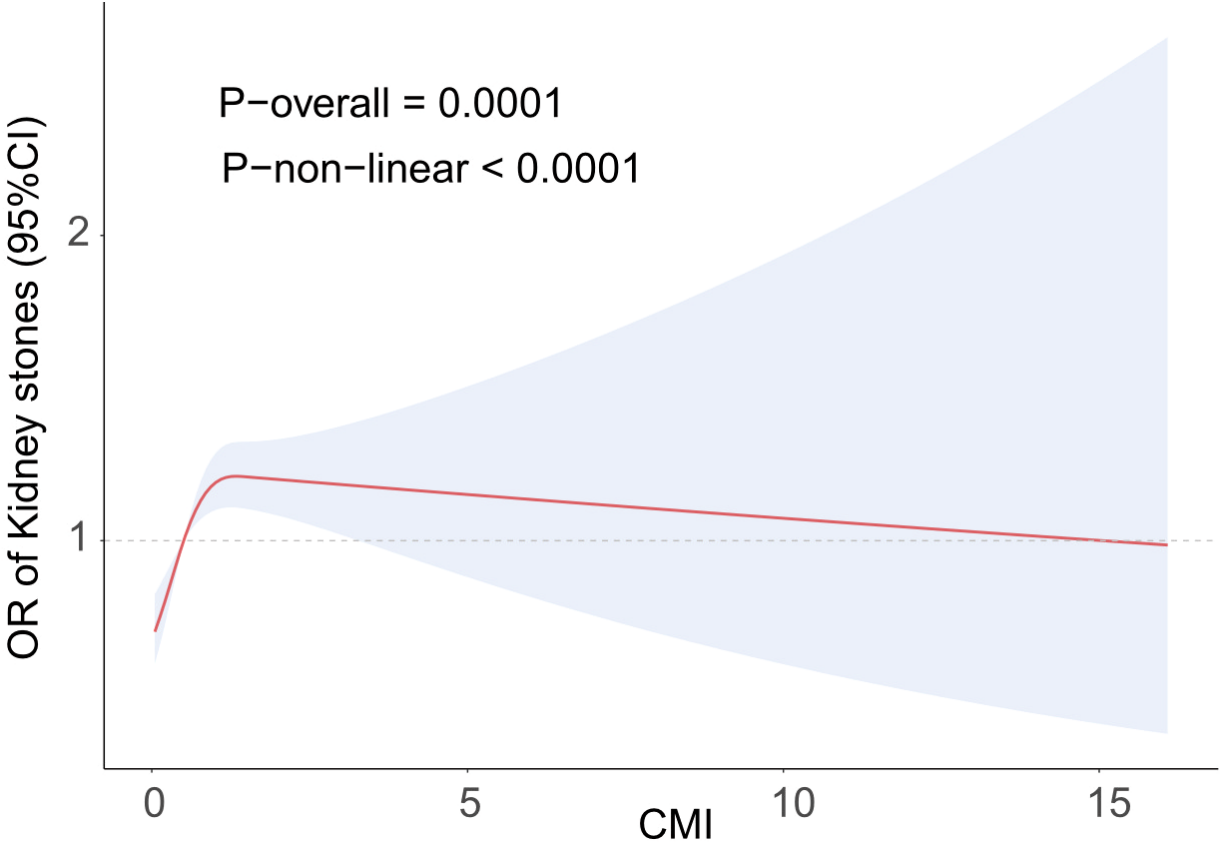
The RCS curve fits the association of CMI with kidney stones. Adjusted for Age, Sex, Race, BMI, PIR, Educational level, Smoke, Activity status, CAD, CKD, Diabetes, SUA, BMI, TC.

**Figure 2** illustrates a nonlinear positive association between kidney stones and CMI, as shown by the RCS analysis. The RCS analysis determined a crucial threshold at CMI=1.34, which was then transformed into a binary variable. **Table 3** displays the results of the segmented logistic regression study. For every unit increase in CMI on the left side of the threshold, there was a 29% increase in the prevalence of kidney stones (OR=1.29, 95% CI=1.09-1.53). However, there was no statistically significant increase in the CMI of kidney stones on the right side of the threshold. There is a clear threshold effect seen in the positive association between CMI and renal stones.

**Table 3:** Two-stage logistic regression between CMI and kidney stones.

	CMI	OR (95%CI) P value
Kidney stones	Standard linear model	1.02 (0.97, 1.06) 0.400
	CMI < 1.34	1.29 (1.09, 1.53) 0.003
	CMI > 1.34	0.99 (0.92, 1.03) 0.500
	Log-likelihood ratio test	0.004

CMI, Cardiometabolic index; OR, Odds ratio; CI, Confidence interval.

Adjusted for Age, Sex, Race, BMI, PIR, Educational level, Smoke, Activity status, CAD, CKD, Diabetes, SUA, BMI, TC.

### 3.4 Subgroup analysis

Subgroup analyses were implemented to investigate the potential influence of log CMI on kidney stones in a diverse array of populations. **Table 4** indicates that the association between CMI and kidney stones was highly significant in the following populations: female, obese, diabetic and CKD. This association was observed subsequent to the adjustment of all covariates. An interaction effect with the diabetes factor was identified by the interaction test.

**Table 4:** Subgroup analysis between log CMI and kidney stones.

Characteristic	Group	OR (95%CI) P value	P for interaction
Age	<50	1.22 (1.06, 1.41) 0.006	0.090
	>50	1.12 (0.96, 1.31) 0.140	
Sex	Male	1.16 (0.99, 1.36) 0.069	0.600
	Female	1.22 (1.02, 1.46) 0.026	
Race	Mexican American	1.08 (0.81, 1.44) 0.600	0.200
	Non-Hispanic black	0.95 (0.70, 1.30) 0.800	
	Non-Hispanic white	1.19 (1.04, 1.37) 0.013	
	Others	1.31 (1.03, 1.67) 0.029	
BMI	Underweight	4.00 (0.87, 18.4) 0.073	0.400
	Normal	1.18 (0.89, 1.58) 0.200	
	Obese	1.18 (1.05, 1.33) 0.007	
Diabetes	No	1.15 (1.00, 1.32) 0.046	0.027
	Yes	1.55 (1.17, 2.05) 0.003	
CAD	No	1.17 (1.04, 1.31) 0.009	0.400
	Yes	1.35 (0.97, 1.88) 0.076	
CKD	No	1.15 (1.03, 1.28) 0.014	0.200
	Yes	2.21 (1.30, 3.75) 0.004	

BMI, body mass index; CAD, coronary artery disease; CKD, chronic kidney disease.

### 3.5 Sensitivity analyses

In order to evaluate the reliability and robustness of the findings of this study, we excluded extreme values of CMI ± 3 SD, which left 14039 participants for sensitivity analyses. The results, as illustrated in **Supplementary Table 1**, indicated that the positive association between kidney stone prevalence and CMI was consistent across all models. More specifically, for each 1-unit increase in CMI, a 21% increase in kidney stone prevalence was observed. The reliability of the results of this study was further confirmed by the fact that this positive association remained consistent even after being transformed into a categorical variable.

## 4 Discussion

This study was based on the NHANES systematic study on the association between CMI and kidney stones prevalence. A positive link exists between an elevated CMI and an increased susceptibility to kidney stones in the United States. This association remained true even after controlling for a number of confounding factors. The correlation was more pronounced when the CMI was less than 1.34, as indicated by the RCS results. This indicates that CMI might be an important measure for evaluating the frequency of kidney stones and that tracking CMI could help identify people who are at a significant prevalence of acquiring kidney stones.

Studies have shown a strong association between metabolic issues and the formation and reappearance of kidney stones. A correlation has been shown by researchers between metabolic syndrome and an increased susceptibility to kidney stones (16–19). These researches have shown that the development of kidney stones is closely linked to conditions connected to metabolic syndrome, such as hyperglycemia, obesity, and dyslipidemia (20–24). Additionally, patients with metabolic syndrome frequently demonstrate elevated levels of calcium oxalate in their urine, which elevates the likelihood of stone formation (25–27). Furthermore, Carbone A et al. conducted research that demonstrated a substantial correlation between obesity and kidney stones, primarily through mechanisms associated with metabolic syndrome (24). Obesity can result in hypercalciuria, hyperoxaluria, and hypocitraturia, all of which are risk factors for kidney stones, due to insulin resistance (18, 24, 28). Our research provides additional evidence to substantiate these conclusions and introduces the CMI as a comprehensive metric, providing a more comprehensive assessment method. A number of factors have been linked to kidney stones in the past. These include the weight-adjusted waist circumference index (WWI), the lipid accumulation product index (LAP), the body roundness index (BRI), the body size index (ABSI), and the non-high-density lipoprotein to high-density lipoprotein ratio (NHHR) (14, 29–33). However, there is presently no correlation study between CMI and kidney stones, as CMI includes the WHtR and the TG/HDL-C ratio. In order to more accurately represent the relationship between lipid metabolism and body measurement index, we conducted a study to investigate the relationship between kidney stones and CMI. Our findings, regardless of whether they were continuous or categorical variables, indicated that CMI was significantly associated with kidney stones.

A variety of factors can be attributed to the correlation between kidney stones and CMI. First, increasing CMI levels are associated with hyperglycemia and insulin resistance, which increases excretion of calcium and oxalate in the urine. This then helps the calcium oxalate stones to develop (16). The risk of stones is further heightened by dyslipidemia, which can result in diminished urine concentration and renal tubular injury (such as high TG and low HDL-C) (16). Additionally, metabolic disorders may induce oxidative stress, which is linked to the formation of kidney stones (34). To be more precise, oxidative stress exacerbates renal tubular epithelial cell injury, which in turn increases the probability of oxalate and calcium ion adsorption and crystal formation (35). Chronic inflammation, on the other hand, promotes the release of cytokines, altering the renal microenvironment and facilitating the formation and growth of stones (36). In this study, although we did not conduct experimental investigations on populations with different CMI levels, oxidative stress, as a potential mechanistic link between CMI and kidney stone formation, remains a valuable area for further exploration. Oxidative stress has been shown to play a critical role in various metabolic abnormalities and renal damage. By generating reactive oxygen species (ROS), it damages cells, triggers inflammatory responses, and alters metabolic processes, making it a key mechanism in kidney stone formation (37). In individuals with higher CMI levels, poor metabolic health may be accompanied by elevated oxidative stress, thereby increasing the risk of kidney stones. Future studies could recruit populations with varying CMI levels and use oxidative stress biomarkers to measure changes in oxidative stress levels and explore their association with kidney stone development. These biomarkers would help assess the degree of oxidative stress in individuals, providing stronger support for causal inference.

The association between CMI and kidney stones was more pronounced in specific populations, such as females, individuals under the age of 50, and those with obesity, CKD, CAD, and diabetes, as revealed by subgroup analyses. These results indicate that certain subgroups may be more susceptible to fluctuations in CMI, which could elevate the likelihood of kidney stones. In our analysis, the non-Hispanic white population exhibited a stronger association between CMI and kidney stones, which may be closely related to the prevalent metabolic characteristics within this group. Firstly, obesity is a common metabolic issue among non-Hispanic whites (38), and it is often accompanied by insulin resistance, dysregulated fat metabolism, and chronic low-grade inflammation. Research has shown that obesity promotes kidney stone formation not only by altering hormone levels, such as insulin and adipokines, but also by affecting urinary composition, such as increased levels of urinary calcium, oxalate, and uric acid (24). Additionally, metabolic syndrome induced by obesity, including conditions such as hyperglycemia, dyslipidemia, and hypertension, may further enhance the sensitivity of CMI to kidney stone formation (39). The correlation is more pronounced in individuals under the age of 50, which may be attributed to a variety of factors. Firstly, metabolic function decreases with age, leading to an increased prevalence of metabolic disorders. Second, chronic diseases are more prevalent in the older population and are strongly associated with kidney stone formation. The association between kidney stones and CMI may be weakened by these factors. Especially after the age of 50, metabolic flexibility gradually declines, increasing the risk of metabolic dysregulation, which may lead to elevated CMI and exacerbate the prevalence of kidney stone formation. This finding also suggests that interventions targeting CMI may be more effective in younger populations, particularly in the early stages when metabolic flexibility has not yet significantly declined. By improving metabolic health, such as increasing physical activity and enhancing dietary habits, younger individuals can maintain a healthier metabolic state, thereby reducing the risk of elevated CMI and lowering the likelihood of kidney stone formation.

The hormonal and metabolic profiles of female are distinct from those of males. For example, changes in hormone levels before and after menopause can affect lipid and glucose metabolism, altering the association between CMI and kidney stones (40, 41). Additionally, women typically have a greater body fat percentage, which may further exacerbate the impact of metabolic disorders (42). The correlation is more pronounced in chronic renal populations and may be due to decreased renal function thereby reducing the ability to concentrate urine and increasing the concentration of stone-forming substances in the urine, thereby promoting stone formation. In the female population, particularly among postmenopausal women, hormonal changes have a profound impact on metabolic health. After menopause, the sharp decline in estrogen levels often leads to a range of metabolic disturbances, including dyslipidemia, insulin resistance, and calcium metabolism imbalance (43). These metabolic issues directly affect changes in CMI and may increase the risk of kidney stone formation through various physiological pathways. A classic metabolic disorder, diabetes is distinguished by insulin resistance and hyperglycemia (44). The prevalence of kidney stones formation may be elevated in diabetic patients with high CMI values, which may suggest a more severe metabolic imbalance. Moreover, participants without CAD exhibited a stronger association between CMI and kidney stones, which may be related to a range of metabolic disturbances commonly seen in CAD, such as hyperglycemia and dyslipidemia. These factors can directly influence kidney stone formation, complicating the underlying mechanisms (45). Additionally, CAD patients often receive a variety of treatments, including antihypertensive drugs, statins, and antiplatelet medications, which, in some cases, may affect renal function or urinary composition, potentially diminishing the association between CMI and kidney stones. In contrast, individuals without CAD may be more susceptible to changes in CMI. This finding suggests that there may be distinct metabolic backgrounds and therapeutic interventions between CAD and non-CAD patients, warranting further investigation in future studies.

The validity of the findings is improved by the use of the NHANES database, which provides a large, representative dataset. CMI, as a comprehensive indicator of cardiovascular metabolic health, reflects the status of multiple metabolic pathways in the body, such as lipid metabolism, insulin sensitivity, and energy balance. In individuals with obesity and diabetes, CMI levels are often elevated due to the presence of metabolic abnormalities and associated risk factors. These populations generally face a higher prevalence of kidney stone formation. Therefore, early monitoring of CMI could provide valuable insights for public health screening. By screening for CMI in these high-risk groups, potential metabolic issues and the prevalence of kidney stones can be identified early, offering data support for the development of personalized prevention and intervention strategies.

However, this study is subject to several limitations. Firstly, causal relationships cannot be determined due to the cross-sectional design. Secondly, although multiple confounding factors were controlled for, there may still be unmeasured confounders influencing the results, such as dietary patterns, genetic predispositions, recall bias, and mental health factors. The causal association between kidney stones and CKD could be further validated in future studies through longitudinal study designs. Additionally, experimental studies would help elucidate the specific molecular mechanisms through which CMI influences kidney stone formation, thereby providing new targets for prevention and treatment.

## 5 Conclusion

This study illustrated a substantial association between CMI and an elevated prevalence of kidney stones, underscoring the significance of CMI as a comprehensive indicator for metabolic risk assessment. Additionally, it suggested that monitoring CMI levels could be beneficial in identifying populations with a high prevalence of kidney stones.

## Data Availability

Publicly available datasets were analyzed in this study. This data can be found here: https://www.cdc.gov/nchs/nhanes/nhanes_products.htm.
