# Supplementary material for: Associations between cardiometabolic index with kidney stones: evidence from NHANES 2007-2018
Source: Front Endocrinol (Lausanne). 2025 Mar 10;16:1485477. doi: 10.3389/fendo.2025.1485477 (PMC11930802; doi:10.3389/fendo.2025.1485477)
Supplement: Supplementary file 1 [file Table1.docx]

Supplementary Material

**Supplementary Table 1 Sensitivity analysis of CMI and prevalence of kidney stones.**

|  |  | Model 1  OR (95%CI) P value | Model 2  OR (95%CI) P value | Model 3  OR (95%CI) P value |
| --- | --- | --- | --- | --- |
| Kidney stones | log CMI | 1.44 (1.31, 1.59) <0.001 | 1.36 (1.23, 1.51) <0.001 | 1.21 (1.07, 1.36) 0.003 |
|  | Q1 | [Reference] | [Reference] | [Reference] |
|  | Q2 | 1.41 (1.09, 1.84) 0.010 | 1.33 (1.02, 1.72) 0.033 | 1.17 (0.88, 1.56) 0.300 |
|  | Q3 | 1.88 (1.45, 2.44) <0.001 | 1.73 (1.32, 2.25) <0.001 | 1.41 (1.04, 1.92) 0.029 |
|  | Q4 | 2.17 (1.71, 2.76) <0.001 | 1.91 (1.49, 2.44) <0.001 | 1.47 (1.09, 1.98) 0.012 |
|  | P for trend | <0.001 | <0.001 | 0.013 |

CI: Confidence interval; CMI: Cardiometabolic index; OR: Odds ratio; Q: Quartiles

Model 1: No covariates adjusted; Model 2: Adjusted for Age, Sex, and Race; Model 3: Adjusted for Age, Sex, Race, BMI, PIR, Educational level, Smoke, Activity status, CAD, CKD, Diabetes, SUA, BMI, TC.
